# Can Salivary Innate Immune Molecules Provide Clue on Taste Dysfunction in COVID-19?

**DOI:** 10.3389/fmicb.2021.727430

**Published:** 2021-10-11

**Authors:** Aaron Ermel, Thankam Paul Thyvalikakath, Tatiana Foroud, Babar Khan, Mythily Srinivasan

**Affiliations:** ^1^Division of Infectious Diseases, Department of Internal Medicine, Indiana University Purdue University at Indianapolis, Indianapolis, IN, United States; ^2^Indiana University School of Medicine, Indiana University Purdue University at Indianapolis, Indianapolis, IN, United States; ^3^Department of Cariology, Operative Dentistry and Dental Public Health, Indiana University Purdue University at Indianapolis, Indianapolis, IN, United States; ^4^Indiana University School of Dentistry, Indiana University Purdue University at Indianapolis, Indianapolis, IN, United States; ^5^Regenstrief Institute, Inc., Indianapolis, IN, United States; ^6^Department of Medical and Molecular Genetics, Indiana University Purdue University at Indianapolis, Indianapolis, IN, United States; ^7^Division of Pulmonary, Critical Care, Sleep and Occupational Medicine, Department of Medicine, Indiana University School of Medicine, Indianapolis, IN, United States; ^8^Department of Oral Pathology, Medicine and Radiology, Indiana University Purdue University at Indianapolis, Indianapolis, IN, United States

**Keywords:** saliva, COVID-19, innate, receptor, taste, health informatics

## Abstract

Emerging concerns following the severe acute respiratory syndrome coronavirus-2 (SARS-CoV2) pandemic are the long-term effects of coronavirus disease (COVID)-19. Dysgeusia in COVID-19 is supported by the abundant expression of the entry receptor, angiotensin-converting enzyme-2 (ACE2), in the oral mucosa. The invading virus perturbs the commensal biofilm and regulates the host responses that permit or suppress viral infection. We correlated the microbial recognition receptors and soluble ACE2 (sACE2) with the SARS-CoV2 measures in the saliva of COVID-19 patients. Data indicate that the toll-like receptor-4, peptidoglycan recognition protein, and sACE2 are elevated in COVID-19 saliva and correlate moderately with the viral load.

## Introduction

The coronavirus disease (COVID-19) pandemic caused by the novel severe acute respiratory syndrome coronavirus-2 (SARS-CoV2) has taken a toll on global health and economy, infecting 197 million people and causing over four million deaths worldwide as of July 30, 2021. While most individuals with symptomatic illness recover completely by 4 weeks, a significant proportion of individuals experience long-term symptoms (Centre for disease control and prevention, 2021). Fatigue, headaches, difficulties in concentrating/mental fog, change/loss of taste, change/loss of smell, sleep disorders, and dyspnea constitute the most frequent symptoms in long COVID-19 (Estiri et al., 2021). The SARS-CoV2 virus encodes four major structural proteins, including the spike protein, the membrane protein, the envelope protein, and the nucleocapsid protein. The spike protein of the viral envelope binds the angiotensin-converting enzyme 2 (ACE2) on the surface of target cells for host cell entry. Following ACE2 binding, the spike protein is cleaved by the host protease, transmembrane protease serine 2 (TMPRSS-2), facilitating the intracellular release of viral RNA. A second host protease, the tumor necrosis factor α-converting enzyme (TACE), cleaves the ACE2 protein at the transmembrane helix and releases the ectodomain as a soluble protein (sACE2) (Heurich et al., 2014).

Complex symbiotic relationships between members of the commensal microflora, which is site specific, contribute to the maintenance of human health (Li et al., 2019). The host microbiome could either be neutral or hinder or facilitate viral infection (Wilks et al., 2013). Perturbations in microbiome following viral invasion have been shown to trigger host innate immune responses by signaling *via* pattern recognition receptors such as toll-like receptors (TLR)—for example, while inflammation and cytokine secretion induced by the influenza virus promotes pneumococcal infection, co-infection with *Echinacea purpurea* suppresses influenza A infection by inhibiting TLR-4 and NF-κB activation (Wilks et al., 2013). In COVID-19, a review of clinical case series suggests that bacterial co-infection or superinfection correlates positively with disease severity and poor outcomes (Feldman and Anderson, 2021). Pertinently, emerging evidence suggests that, in addition to ACE2, the SARS-CoV2 could use TLR-2 or TLR-4 for host cell entry (Gadanec et al., 2021). Furthermore, the observations of TLR upregulation by the damage-associated molecular patterns in COVID-19 clinical samples as well as the proposed models of direct binding of SARS-CoV2 spike protein with TLRs1, 4, and 6 suggest specific roles for these TLRs in facilitating viral entry and/or pathology (Sohn et al., 2020; Gadanec et al., 2021). Among the TLRs, TLR-4 has been shown to exhibit the strongest protein–protein interaction with the spike protein of COV-2 (Choudhury and Mukherjee, 2020). Furthermore, it has been suggested that the CoV2/TLR-4 binding induced an increased expression of interferon-stimulated gene expression, and the resultant upregulation of ACE-2 represents a mechanistic link between TLR-4 and CoV2 cellular entry (Gadotti et al., 2020).

Much like the nasopharynx, the oral mucosa is also a portal for entry of airborne virus, including SARS-CoV2. The oral epithelial cells have been shown to express abundantly the SARS-CoV2 receptor, ACE2, and the associated metalloproteases TMPRSS-2 and TACE (Hirayama et al., 2017; Srinivasan et al., 2020). Additionally, the expression of TLR-2 and TLR-4 in the oral epithelium could contribute to SARS-CoV2 entry (Gadanec et al., 2021). The virus has been consistently detected in saliva, and some studies have suggested that the SARS-CoV2 load is higher in saliva than that in the nasopharyngeal swabs. Since the SARS-CoV2 and host interactions within the oral cavity could contribute to the symptoms and the pathology of COVID-19, we hypothesized that the pattern recognition receptors that recognize the oral flora will be upregulated in SARS-CoV2-infected individuals.

Whole saliva is a mixture of secretions (from salivary glands, passive diffusion from blood, and from cells lining the oral mucosa) and cellular components that include corpuscles, exfoliated epithelial cells, and the oral microbiome. Hence, salivary proteome and transcriptome are recognized as excellent resources for investigating the markers for oral and systemic diseases (Lee et al., 2011). Previously, we and others have reported that the salivary TLR-2 and TLR-4 are upregulated in inflammatory diseases of the oral cavity. Here we assessed the expression of TLR-2, TLR-4, and peptidoglycan recognition protein (PGRP)-4 and evaluated the association of changes in these markers as it related to SARS-COV-2 infection in the saliva of hospitalized patients with COVID-19.

## Methods

### Study Cohort and Samples

The study cohort included 30 symptomatic, non-ventilated patients with COVID-19 diagnosed by RT-PCR SARS-CoV2 testing from nasopharyngeal swabs and admitted to the IU Health hospital system between September 2020 and December 2020. Unstimulated whole saliva (UWS) was collected within 48 h of admission to the hospital with the help of the Indiana Biobank and in accordance with the institutional review board (IRB protocol # 1105005445). The UWS was collected using the ORA-100 ORAcollect RNA kit following the instructions of the manufacturer (DNA Genotex, ON, Canada). The control UWS included archived saliva bank samples from the salivary research laboratory at Indiana University School of Dentistry that predated the COVID-19 pandemic. All UWS samples were processed at the Infectious Disease Laboratory of the Indiana University School of Medicine. SARS-CoV2 was detected qualitatively by RT-PCR amplifying the RdRp and N genes on the Abbott m2000 Real Time System (Abbott Laboratories, Chicago, IL, United States). Quantitative viral load testing was performed by RT-qPCR targeting the N-gene utilizing the N1 primers from the 2019-nCoV CDC kit against a six-point standard.

The sACE2 activity in each UWS was determined using the CoviDrop^TM^ SARS-CoV-2 Spike-ACE2 binding activity/inhibition assay kit (Epigentek, Farmingdale, NY, United States). The binding activity was calculated according to the equation binding activity = (sample OD− blank OD)/(slope × sample volume, μl) × 1,000, where OD is optical density, blank is the negative control, and slope is calculated from the linear regression of ACE2 standards of known concentration.

The saliva RNA was reverse-transcribed using the iScript cDNA synthesis kit (Biorad, Austin, TX, United States), and an equal quantity of cDNA was amplified for TLR-2, TLR-4, PGPR-4, IFN-γ, and β-actin as a house-keeping gene using the SYBR green/ROX qPCR master mix (SA Biosciences, Frederick, MD, United States) on ABI PRISM^TM^ 7000 Sequence Detection Systems (Thermo Fisher Scientific, United States). The primers used are as follows: βactin-F: 5′-GCCAACCGCGAGAAGATGA-3′, βactin-R: 5′-CATCACGATG CCAGTGGTA-3′; TLR-2F: 5′-ACCTGTGTGACTCTCCATCC-3′, TLR 2R: 5′-GCAGCATCA TTGTTCTCTC-3′; TLR-4F: 5′-TTCCTCTCCTGCGTGAGAC -3′, TLR-4R: 5′-TTCATAGGGTTCAGGGACAG-3′; IFN-γ: 5′-AGTGCCAGCAGAAATATCCTCC-3′, IFNγR: 5′-GAACG AGGTGAGCCAAATATCC-3′, and peptidoglycan recognition protein (PGRP)-4F: 5′-TTTTGCCCTCCTCCCCTGCCA-3′, PGRP-4R: 5′-ATGAGGTTTGGAGGCCCTTGG-A-3′. The relative expression (Ct value) of the TLR-2, TLR-4, PGRP-4, and IFN-γ gene was normalized with βactin by subtracting the latter from the former. Because the level of gene expression is inversely proportional to the Ct and the amount of the product doubles with each cycle of the RT-PCR, fold change in the gene expression was calculated by the 2^–Δct^ method.

### Statistics

Differences in sACE2 binding activity and in the measures of the transcripts amplified between the COVID-19 and control samples were determined by Student’s *t*-test and Mann–Whitney *U*-test. The value of *p* < 0.05 was considered significant. A correlation between the measures of SARS-CoV2 and the sACE2 or the transcripts in saliva was determined by Spearman correlation.

## Results

### Clinical Data of the COVID-19 Cohort

**FIGURE 1:**
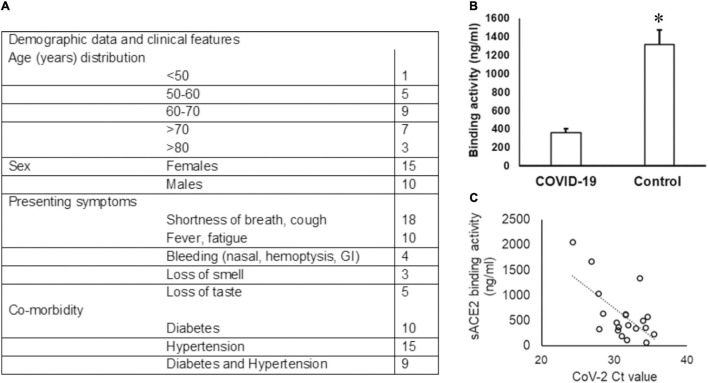
Demographic data of the COVID-19 cohort and sACE2 in saliva. **(A)** The age and sex distribution as well as the presenting symptoms of the COVID-19 cohort included in the study are provided. **(B,C)** The sACE-2 is lower in COVID-19 saliva. **(B)** Unstimulated whole saliva samples collected from hospitalized COVID-19 patients were assessed for sACE2 by ELISA as described in the “Methods” section; *p < 0.05. **(C)** Spearman correlation (*r*_s_) for the relationship between the sACE-2 and the SARS-CoV2 ct value.

Among the 30 COVID-19-positive individuals recruited for this study, unstimulated whole saliva samples from 25 individuals were assessed, as five samples were discarded due to contamination and insufficient collection. The highest incidence of COVID-19 was observed in the seventh decade (36%) of life and a female to male ratio of 1.5:1 in our cohort (see Figure 1A). Shortness of breath was the most common symptom (72%), and 20% of individuals experienced loss of taste.

### SARS-CoV2 and Its Receptor in COVID-19 Saliva

The Ct values for SARS-CoV2 in the UWS samples ranged from 13.60 to 30.47 based on the CN value reported by the Abbott SARS-CoV-2 Real Time Assay. The viral load was calculated in nine samples and ranged from 1.6 to 678 copies/ml. We quantitated the salivary sACE2 binding to the plate-bound spike protein as a measure of sACE2 concentration (Figure 1A). The sACE-2 was significantly lower in the UWS of COVID-19 patients than that in the control cohort (Figure 1B). Interestingly, the sACE2 exhibited a strong correlation to the viral load (*r*_s_ = −0.5 with the SARS-CoV2 Ct value) (Figure 1C). Significantly, in the nine samples with viral load available, there was a strong positive correlation with sACE2, *r*_s_ = 0.9. Although the sample numbers are few, these observations support reports of potential oral mucosal infection by SARS-CoV2 (Figure 1C).

### Pattern Recognition Receptors in COVID-19 Saliva

**FIGURE 2:**
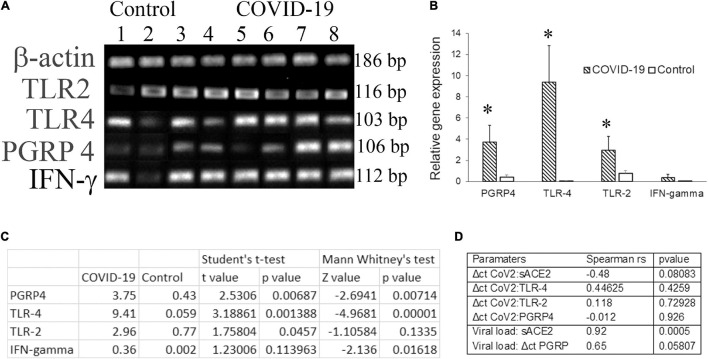
The transcripts for pattern recognition receptors are higher in COVID-19 saliva. Unstimulated whole saliva (UWS) collected from hospitalized COVID-19 patients and archived UWS were assessed for the indicated gene. **(A)** Gel electrophoresis images of the genes of representative samples. **(B)** Mean gene expression normalized to beta actin. **(C)** The table shows the *t*-value, *z*-value, and *p*-value for each transcript assessed. **(D)** The table shows the Spearman correlation (rho) value for the pairs of parameters indicated. *p < 0.05.

Molecular docking studies have demonstrated significant binding of the native spike protein of SARS−CoV−2 to TLR1, TLR4, and TLR6, with TLR4 exhibiting the strongest interaction (Choudhury and Mukherjee, 2020). Previously, inflammation by a respiratory virus such as the influenza virus has been shown to enhance host receptors that either facilitate or suppress bacterial co-infection and modulate the disease (Keynan et al., 2011). Several studies have investigated the transcriptome of cell-free and supernatant saliva for biomarkers of oral and systemic diseases (Lee et al., 2011). Here we investigated the salivary transcripts of microbial pattern recognition receptors that recognize the normal oral flora in COVID-19 patients. The fold change or relative expression of TLR-2, TLR-4, and PGRP-4 was significantly higher in COVID-19 saliva as compared to that in the control saliva (Figures 2A–C).

Dysregulated immune responses and elevated cytokines, in particular, the higher levels of IFN-γ, were related to a poorer prognosis in COVID-19 (Gadotti et al., 2020). Since our cohort included hospitalized COVID-19 individuals, we measured IFN-γ in saliva samples. The fold change in IFN-γ was higher in COVID-19 saliva than that of control saliva, although the difference did not reach a significant level. While the Δct of PGRP-4 exhibited a strong correlation (*r*_s_ = 0.65) with the viral load, that of TLR-4 exhibited a moderate correlation (*r*_s_ = 0.44) with the ct value of CoV2 in COVID-19 saliva (Figure 2D). No definitive correlation was observed between the CoV2 measures and TLR-2 or IFN-γ transcripts (Figure 2D).

## Discussion

Saliva is an excellent biospecimen not only for the detection of SARS-CoV2 but also for the identification of biomarkers of disease severity in COVID-19 (Silva et al., 2021). Here we observed that the CoV2 load is directly correlated with the concentration of sACE2 and that the innate immune and inflammatory markers are upregulated in the saliva of hospitalized COVID-19 patients.

Membrane-bound ACE2 is the port of entry for SARS-CoV2 into the target cells. Several human tissues express ACE2, and the density of expression contributes to the diverse clinical symptoms of COVID-19 (Heurich et al., 2014; Gadanec et al., 2021). A positive feedback loop between the SARS-CoV2-bound ACE2 endocytosis, ACE2 ectodomain shedding, and the associated inflammation-induced ACE2 expression in bystander cells leads to sustained tissue injury in COVID-19. Elevated plasma sACE2 activity that remained high for over 3 months has been reported in symptomatic COVID-19 patients (Patel et al., 2021). We and others have shown that Ace-2 is expressed in the lining mucosa of the oral cavity, including the dorsum of the tongue. The high expression of ACE2 has also been reported in the glandular and ductal epithelial cells of the salivary glands (Pascolo et al., 2020). These observations suggest the active infection of oral mucosa by SARS-CoV2 and support the clinical symptoms of xerostomia and dysgeusia in COVID-19.

Our observation of reduced sACE2 in COVID-19 patients is seemingly paradoxical since SARS-CoV-2 infection of oral epithelial cells should increase TACE-mediated cleavage of the extracellular domain of ACE2. Since we investigated the saliva of hospitalized COVD-19 patients and there is considerable evidence that the SARS-CoV2 load is higher, if not equivalent, to that in the nasopharyngeal swabs, the reduced sACE2 could be attributed to quenching by the virus. Pertinently, the sACE2 has been shown to bind the spike protein of CoV2, and recombinant sACE2 has been suggested as a potential therapeutic for COVID-19 (Monteil et al., 2020).

Together with the nasopharynx, the oral mucosa is the site of first encounter for SARS-CoV2. The oral epithelial cells at the host/viral interface respond to the exposure by chemokine secretion, upregulating TLR-mediated signaling, increasing inflammatory cytokines, and accelerating exfoliation. Transcripts from cellular sediments and cell-free clarified saliva have been assessed for biomarkers for oral and systemic diseases (Lee et al., 2011). Previously, we have shown that the TLR-4 transcript is elevated in chronic oral mucosa inflammatory conditions (Swaminathan et al., 2013). Our observations of higher TLR-4 transcript in the saliva of COVID-19 patients is in alignment with its potential role as a receptor for SARS-CoV2. In this context, it is intriguing to note that TLR-4 signaling and elevated IFN-γ in taste bud epithelial cells have been associated with dysgeusia (Wang et al., 2007).

A recent preprint has reported that the microbiome is distinctly different in COVID-19 saliva samples with a disproportionate increase in select bacterial species (*Veillonella*, *Prevotella*, and *Capnocytophaga*) that are recognized by the peptidoglycan recognition protein including PGRP4 (Iebba et al., 2020). In this context, it is pertinent to note that the transcript for PGRP-4 was higher in the COVID-19 saliva than that in the control samples.

## Conclusion

The CDC and the COVID-19-Associated Hospitalization Surveillance Network (COVID-NET) report that a significant proportion of individuals experience a variety of post-COVID conditions, including long-term smell and taste dysfunctions (Centre for disease control and prevention, 2021). Pathologically, this could be attributed to either a persistent viral load (albeit at a low titer) or progressive cellular/functional alterations. The oral epithelial expressions of ACE2 and TLR-4 support their function as potential reservoirs of CoV-2. Interestingly, the specialized sensory taste bud epithelial cells in the dorsum of the tongue express TLR-4, and inflammation has been shown to activate IFN-γ signaling in these cells. Our data demonstrate an association of an increase in specific microbe recognition receptors and increasing SARS-CoV-2 viral load relative to the control saliva. The limitation of this study is the small sample size and the lack of a control group of individuals who are hospitalized for reasons other than COVID-19. However, this study does demonstrate that these measurements can be performed from saliva in a prospective manner and warrant a further study of the interaction between SARS-CoV-2 and the inflammasome of the oral cavity.

## Data Availability Statement

The original contributions presented in the study are included in the article/supplementary material, further inquiries can be directed to the corresponding author/s.

## Ethics Statement

The studies involving human participants were reviewed and approved by Institutional Review Board, Indiana University Purdue University at Indianapolis. The patients/participants provided their written informed consent to participate in this study.

## Author Contributions

MS: concept, analysis, and writing. TT: concept and review. AE: sample testing, analysis, and review. BK: review and discussion. TF: sample collection and review. All authors contributed to the article and approved the submitted version.

## Conflict of Interest

The authors declare that the research was conducted in the absence of any commercial or financial relationships that could be construed as a potential conflict of interest.

## Publisher’s Note

All claims expressed in this article are solely those of the authors and do not necessarily represent those of their affiliated organizations, or those of the publisher, the editors and the reviewers. Any product that may be evaluated in this article, or claim that may be made by its manufacturer, is not guaranteed or endorsed by the publisher.
